# Health profile and metal exposure over three years in a child cohort study, Bruminha Project

**DOI:** 10.11606/s1518-8787.2025059006931

**Published:** 2025-11-17

**Authors:** Carmen Ildes Rodrigues Fróes Asmus, Maíra Lopes Mazoto, Nataly Damasceno de Figueiredo, Herling Gregorio Aguilar Alonzo, Renan Duarte dos Santos Saraiva, Aline de Souza Espindola Santos, Ana Paula Natividade de Oliveira, Ivisson Carneiro Medeiros da Silva, Michele Alves Costa, Leiliane Coelho André, Sérgio Viana Peixoto, Volney de Magalhães Câmara

**Affiliations:** I Universidade Federal do Rio de Janeiro Instituto de Estudos de Saúde Coletiva Rio de Janeiro RJ Brasil Universidade Federal do Rio de Janeiro. Instituto de Estudos de Saúde Coletiva. Rio de Janeiro, RJ, Brasil; II Universidade Estadual de Campinas Faculdade de Ciências Médicas Campinas SP Brasil Universidade Estadual de Campinas. Faculdade de Ciências Médicas. Campinas, SP, Brasil; III Fundação Oswaldo Cruz Escola Nacional de Saúde Pública Rio de Janeiro RJ Brasil Fundação Oswaldo Cruz. Escola Nacional de Saúde Pública. Rio de Janeiro, RJ, Brasil; IV Universidade Federal de Minas Gerais Faculdade de Farmácia Belo Horizonte MG Brasil Universidade Federal de Minas Gerais. Faculdade de Farmácia. Belo Horizonte, MG, Brasil; V Fundação Oswaldo Cruz Instituto René Rachou Belo Horizonte MG Brasil Fundação Oswaldo Cruz. Instituto René Rachou. Belo Horizonte, MG, Brasil

**Keywords:** Effects of Disasters on Health, Cohort Studies, Metals, Child Health

## Abstract

**OBJECTIVE:**

To describe the health profile and patterns of exposure to mining waste over the period of three years in a cohort of children living in areas affected by the collapse of a mining tailings dam in Brumadinho, Minas Gerais State, Brazil, in 2019.

**METHODS:**

This prospective cohort study included all children aged 0 to six years residing in four selected locations: three in the disaster zone (Parque da Cachoeira, Córrego do Feijão, and Tejuco – exposed areas) and one located 10 km away (Aranha – non-exposed area). Assessments included anthropometric growth, neuromotor and cognitive development, and respiratory conditions. Urine samples were collected and analyzed for lead, arsenic, cadmium, mercury, and manganese using inductively coupled plasma mass spectrometry (ICP-MS).

**RESULTS:**

The average percentage of children assessed in the period was 80% of the projected population. There was an increase in the percentage of children with urinary arsenic levels above the reference value over the three-year period (2021 = 42%; 2022 = 44%; 2023 = 57%), as well as in detection rates of lead (88.9%) and mercury (63.9%) in 2021, both reached 100% in 2023. The median urinary arsenic concentration increased from 2021 (9.35 μg/g; IQR = 5.45–13.9) to 2023 (10.8 μg/g; IQR = 7.0–15.3) in the total sample of children (p = 0.064), with a statistically significant increase among those living in exposed areas (p = 0.015; Parque da Cachoeira). Over the three-year period, there was a decrease in the percentage of neurodevelopmental alterations and overweight/obesity, and an increase in reports of respiratory alterations in the total population, although these changes were not related to metal exposure.

**CONCLUSION:**

The findings indicate ongoing exposure of the metals analyzed in the study population; however, no associated health effects have been identified thus far. It is essential that the Brazilian Unified Health System be structured according to specificities of the productive processes in each territory.

## INTRODUCTION

On January 25, 2019, the collapse of Tailings Dam I at the Córrego do Feijão Mine in the city of Brumadinho, Minas Gerais State, Brazil, released 12 million/m^3^ of mining waste. The disaster caused at least 272 deaths, affected 18 municipalities, compromised the viability and water supply of the Paraopeba River Basin, and contaminated soil and food production^1^.

In June 2019, the Brazilian Ministry of Health proposed the implementation of an Integrated Health Actions Program in Brumadinho, aimed at monitoring the population exposed to the impacts of this disaster, identifying knowledge gaps regarding its medium- and long-term health effects, and supporting the Brazilian Unified Health System (SUS) in responding to these challenges^2^. The program comprises two longitudinal studies launched in 2021: the Brumadinho Health Project, coordinated by the Oswaldo Cruz Foundation of Minas Gerais, and the Bruminha Project, coordinated by the Universidade Federal do Rio de Janeiro (UFRJ). Both involve annual assessments of the affected population over a five-year period^2^.

The *Projeto Bruminha – Estudo Longitudinal da Saúde Infantil em Brumadinho* (Bruminha Project – Longitudinal Study on Child Health in Brumadinho) is a prospective cohort involving children aged 0 to 6 years, residing in four selected locations. It investigates the impacts of the disaster on child health by assessing the occurrence of anthropometric and neurodevelopmental alterations, respiratory disorders, and patterns of exposure to metal residues. This study presents the health profile and waste exposure data collected over the first three years of the cohort.

## METHODS

The Bruminha Project protocol is described by Asmus et al.^3^

### Study Area

The study area of the Bruminha Project consists of four locations. Córrego do Feijão (CF) and Parque da Cachoeira (PC) were directly affected, with residences close (1.5 km) to the mining tailings mud. Tejuco (TJ) was included due to its geographical position downstream from a mining area. Aranha (AR), located over 10 km away from the tailings flow, was considered outside the disaster area.

### Study Population

All children residing in the selected locations up to the age limit of six years and 11 months in 2021, 2022, and 2023 were invited to participate in the project. Considering the situation of interest, in which exposure conditions may change over time, the cohort was designed as a dynamic population, with participants entering and/or leaving throughout the follow-up period. The projected study population was identified based on local residency records provided by the Municipal Health Department.

### Instruments, Data Collection, and Urine Sample Collection

1. Socio-environmental questionnaire: *per capita* income; maternal age and education level; child’s race/skin color, sex, and age; sanitation; and source of drinking water.
2. Anthropometric assessments: weight and height^4^.
3. Clinical form: parental recall of respiratory conditions in children—cough, wheezing, shortness of breath, nasal congestion/runny nose, rattling sounds/discharge, recurrent sneezing, and ear pain (15-day recall); pneumonia, asthma/wheezing, bronchitis, rhinitis/sinusitis, respiratory allergies, and otitis (12-month recall).
4. Denver II Developmental Screening Test: this screening tool consists of 125 items representing skills across four developmental domains (personal-social, fine motor-adaptive, language, and gross motor). It is administered using a combination of tasks performed by the child and information provided by caregivers/parents, aiming to verify the child’s abilities in each domain. Based on the child’s performance, responses are classified as: a) “Pass” when the task is properly completed; b) “Fail” when the child is unable to perform the task; c) “No opportunity” when the task is not a common activity in the child’s routine; or d) “Refusal” when the child declines to attempt the task. A task is coded as “Caution” if the child fails a skill that 75% to 90% of children the same age are able to perform, and as “Delay” if the child fails in activities typically completed by 90% or more of children the same age^5^.
5. Children’s urine samples: collected to measure concentrations of arsenic, cadmium, lead, mercury^6^, and manganese^7^.

Reference values (RV) for urinary concentrations of arsenic, cadmium, and mercury were based on values established by Regulatory Standard No. 7 (NR-7, 1994) of the Brazilian Ministry of Labor, whose version in force at the beginning of the study (July 2021) provided RVs for populations not occupationally exposed to these metals^8^. For urinary manganese and lead, references were drawn from the Agency for Toxic Substances and Disease Registry (ATSDR)^9^ and Saravanabhavan et al.^10^

### Laboratory Analysis

Metal concentrations were analyzed using inductively coupled plasma mass spectrometry (ICP-MS). The limit of detection and the limit of quantification for arsenic (total), cadmium, lead, mercury, and manganese were 0.1 μg l-1^11^.

### Statistical Analysis

Sociodemographic characteristics were described for the total study population and by location. For categorical variables, relative frequencies were calculated, and for continuous variables, measures of central tendency and variability were used.

Anthropometric profile, neurodevelopment, and respiratory symptoms were described for the total population and by localities according to the year of evaluation. Anthropometric measurements were categorized based on Body Mass Index (BMI) for the corresponding age group^4^: underweight (< 18.5 kg/height^2^); normal weight (18.6–24.9 kg/height^2^); and overweight/obesity (> 30 kg/height^2^). Respiratory disorders were organized into three categories: upper airway conditions (rhinitis/sinusitis and otitis); lower airway conditions (pneumonia, asthma/wheezing, and bronchitis); and respiratory signs and symptoms (cough, wheezing, shortness of breath, nasal congestion/runny nose, recurrent sneezing, rattling sounds/discharge, and ear pain). Respiratory allergy was assessed separately^12^. Neurodevelopment was categorized into two groups: 1) Denver – normal: children who performed all tasks or had only one “caution” in any the domains; 2) Denver – risk: children with more than one “caution” across domains, or with one or more “delays” in any of the four domains. Statistical differences were assessed using the chi-square test (Yate and Pearson’s continuity correction test).

To analyze metal exposure patterns, detection rates were calculated for each metal, defined as the percentage of urine samples in which the metal was detected relative to the total number of valid urine samples analyzed. Urine samples collected with creatinine concentrations of 0.3 g/L to 3.3 g/L were considered valid.

Median concentrations and interquartile ranges (P25–P75) of urinary metals were established for each location and year of evaluation. To compare concentrations across years in each location, the Kruskal–Wallis test for independent samples was applied to verify differences in variability for each metal.

### Ethical Aspects

This study was approved by the Research Ethics Committee of UFRJ, under No. 5.201.083.

## RESULTS

### Study Population Characteristics

The average percentage of children assessed throughout the three years was 80% of the projected population. In 2021, 2022, and 2023, 42.9%, 33.8%, and 53.4% of the children, respectively, were over four years of age. A higher prevalence of male children and non-White populations (based on maternal reference) was observed, except in 2022 (Table 1).

**Table 1:** Proportion of children assessed by location and sociodemographic characteristics. Bruminha Project, 2021, 2022, and 2023.

Characteristic	Total			Aranha			Córrego do Feijão			Parque da Cachoeira			Tejuco		
	2021	2022	2023	2021	2022	2023	2021	2022	2023	2021	2022	2023	2021	2022	2023
N. assessed^a^	217 (62)	198 (105)	131 (74)	98 (60)	87 (104)	65 (77)	30 (59)	33 (91)	13 (88)	40 (69)	39 (108)	35 (106)	49 (65)	39 (100)	18 (49)
Age - n (%)															
0–11 months	29 (13.3)	41 (20.7)	8 (6.1)	10 (10.2)	11 (12.6)	2 (3.1)	5 (16.6)	9 (27.3)	1 (7.7)	6 (15)	11 (28.2)	3 (8.6)	8 (16.3)	10 (25.6)	2 (11.1)
1–2 years	28 (12.9)	33 (16.7)	21 (16.0)	16 (16.3)	22 (25.3)	8 (12.3)	3 (10)	3 (9.1)	2 (15.4)	5 (12.5)	3 (7.7)	9 (25.7)	4 (8.2)	5 (12.8)	2 (11.1)
> 2–4 years	67 (30.9)	57 (28.8)	32 (24.4)	28 (28.6)	25 (28.7)	16 (24.6)	11 (36.7)	9 (27.3)	4 (30.7)	15 (37.5)	13 (33.4)	7 (20.0)	13 (26.5)	10 (25.6)	5 (27.8)
> 4 years	93 (42.9)	67 (33.8)	70 (53.4)	44 (44.9)	29 (33.4)	39 (60.0)	11 (36.7)	12 (36.3)	6 (46.2)	14 (35.0)	12 (30.7)	16 (45.7)	24 (49.0)	14 (36.0)	9 (50.0)
Total No.	217	198	131	98	87	65	30	33	13	40	39	35	49	39	18
Sex - n (%)															
Female	103 (47.5)	99 (50.0)	55 (42.0)	44 (44.9)	46 (52.9)	30 (46.1)	13 (43.3)	17 (51.5)	5 (38.5)	19 (47.5)	16 (41.0)	13 (37.2)	27 (55.1)	20 (51.3)	7 (38.9)
Male	114 (52.5)	99 (50.0)	76 (58.0)	54 (55.1)	41 (47.1)	35 (53.9)	17 (56.7)	16 (44.5)	8 (61.5)	21 (52.5)	23 (59.0)	22 (62.8)	22 (44.9)	19 (48.7)	11 (61.1)
Total	217	198	131	98	87	65	30	33	13	40	39	35	49	39	18
Ethnicity - n (%)															
White	78 (38.8)	99 (50.0)	47 (36.4)	44 (48.3)	46 (52.9)	29 (45.3)	7 (26.9)	17 (51.5)	3 (27.2)	14 (37.8)	16 (41.0)	8 (22.9)	13 (27.7)	20 (51.3)	7 (38.8)
Non-White	123 (61.2)	99 (50.0)	82 (63.6)	47 (51.7)	41 (47.1)	35 (54.7)	19 (73.1)	16 (48.5)	9 (81.8)	23 (62.2)	23 (59.0)	27 (77.1)	34 (72.3)	19 (48.7)	11 (61.1)
Total^a^	201	198	129	91	87	64	26	33	11	37	39	35	47	39	18
Mother’s mean age															
Mean (SD)	33 (9)	32 (6)	33 (6)	33 (9)	33 (6)	33 (6)	32 (9)	31 (5)	32 (6)	32 (9)	33 (6)	33 (5)	33 (10)	33 (8)	34.8 (6)
Mother’s education in years															
1 to 9 years	58 (33.2)	36 (27.7)	23 (20.4)	30 (37.0)	16 (27.0)	11 (20.0)	3 (14.3)	4 (21.0)	2 (18.2)	12 (33.3)	7 (25.0)	7 (22.6)	13 (35.1)	9 (38.0)	3 (18.7)
> 9 years	117 (66.8)	94 (72.3)	90 (79.6)	51 (63.0)	43 (73.0)	44 (80.0)	18 (85.7)	15 (79.0)	9 (81.8)	24 (66.7)	21 (75.0)	24 (77.4)	24 (64.9)	15 (63.0)	13 (81.3)
Total	175	130	113	81	59	55	21	19	11	36	28	31	37	24	16
Sanitary sewage															
Septic tanks	175 (87.0)	152 (96.8)	113 (89.0)	87 (93.6)	70 (97.3)	55 (88.7)	25 (89.2)	24 (100)	12 (92.3)	36 (94.8)	31 (100)	34 (97.2)	27 (64.3)	27 (90.0)	12 (70.6)
Network	8 (4.0)	5 (3.2)	10 (7.9)	3 (3.2)	2 (2.7)	7 (11.3)	2 (7.2)	0	1 (7.7)	1 (2.6)	0	0	2 (4.7)	3 (10)	2 (11.8)
Open airs/rivers/lakes	18 (9.0)	0	4 (3.1)	3 (3.2)	0	0	1 (3.6)	0	0	1 (2.6)	0	1 (2.8)	13 (31.0)	0	3 (17.6)
Total	201	157	127	93	72	62	28	24	13	38	31	35	42	30	17
Source of water for consumption															
Mineral	125 (60.1)	100 (58.5)	83 (64.3)	24 (25.3)	17 (23.0)	26 (41.9)	28 (100)	25 (92.6)	11 (78.6)	37 (92.5)	32 (94.1)	34 (97.2)	36 (80)	26 (72.2)	12 (66.7)
Other^b^	83 (39.9)	71 (41.5)	46 (35.7)	71 (74.7)	57 (77.0)	36 (58.1)	0	2 (7.4)	3 (21.4)	3 (7.5)	2 (5.9)	1 (2.8)	9 (20.0)	10 (27.8)	6 (33.3)
Total	208	171	129	95	74	62	28	27	14	40	34	35	45	36	18

SD: standard deviation.

^a^ Number of children evaluated and percentage in relation to the predicted total number of children according to the local health unit registration.

^b^ Other sources of drinking water: shared cistern and well or spring.

The mean age of mothers/caregivers each year ranged from 32.4 (6.1) to 33 (9.0) years. An increase was observed in the percentage of mothers/caregivers with more than nine years of education, in total (66.8%–79.6%) and by location, over the three-year period. Septic tanks were the most prevalent type of sanitation across all years of assessment, both in the total number of households and by location. TJ had the highest percentage of households with open sewage disposal (into open airs, rivers, or lakes) in 2021 (31%) and 2023 (17.6%). Across all three years, the main source of drinking water for the total sample, as well as in PC, CF, and TJ, was mineral water. In AR, most households used other sources of water (shared cistern, wells, or springs) rather than mineral water across all years (Table 1).

### Health Profile

Most children had a normal BMI in 2021 (76%), 2022 (83%), and 2023 (91%), with a decrease in percentages of overweight and obesity over this period across all locations studied.

Over the three years, there was a significant reduction in the percentage of children classified as at risk of developmental delay based on Denver II, using 2021 as the reference year. In the total population, this reduction was approximately 34% (p < 0.05) for both 2022 and 2023. When analyzed by location, only AR showed no significant difference in either of the assessed periods. In CF (p = 0.005) and PC (p = 0.040), the occurrence of developmental delay risk decreased by 97% and 48%, respectively, in 2022. In TJ (p = 0.01) a 66% reduction was observed in 2023 when compared with 2021 (Table 2).

**Table 2:** Neurodevelopmental assessment and Body Mass Index classification by year, total population, and location. Bruminha Project, 2021, 2022, and 2023.

Location	Neurodevelopment - n (%)		p^a^	Classification according to BMI - n (%)			p^a^
	Normal	Risk of delay		Underweight	Normal	Overweight/obesity	
Total							
2021	111 (57.5)	82 (42.5)	**-**	0	164 (76.0)	52 (24.0)	-
2022	137 (71.7)	54 (28.3)	**0.003**	3 (1.5)	165 (83.3)	30 (15.2)	**0.025**
2023	86 (71.7)	34 (28.3)	**0.010**	0	119 (90.8)	12 (9.2)	**0.001**
Aranha							
2021	59 (67.8)	28 (32.2)	-	0	72 (74.2)	25 (25.8)	-
2022	59 (68.6)	27 (31.4)	0.95	1 (1.1)	76 (84.4)	13 (15.6)	0.10
2023	40 (71.4)	16 (28.6)	0.64	0	56 (89.0)	7 (11.0)	**0.03**
Córrego do Feijão							
2021	12 (44.4)	15 (55.6)	-	0	25 (83.3)	5 (16.7)	-
2022	25 (83.3)	5 (16.7)	**0.005**	1 (3.3)	26 (83.9)	2 (6.4)	0.50
2023	10 (66.7)	5 (33.3)	0.16	0	13 (86.7)	2 (13.3)	0.9
Parque da Cachoeira							
2021	18 (50)	18 (50)	-	0	23 (57.5)	17 (42.5)	-
2022	28 (73.7)	10 (26.3)	**0.040**	1 (2.6)	31 (81.6)	6 (15.8)	**0.025**
2023	21 (67.7)	10 (32.3)	0.14	0	33 (94.3)	2 (5.7)	**0.001**
Tejuco							
2021	22 (51.2)	21 (48.3)	-	0	44 (89.8)	5 (10.2)	-
2022	25 (18.3)	12 (22.2)	0.13	0	32 (82.0)	7 (18.0)	0.50
2023	15 (83.3)	3 (16.7)	**0.01**	0	17 (94.4)	1 (5.6)	0.75

BMI: Body Mass Index.

^a^ Chi-square test to compare proportions observed each year, with 2021 as a reference.

Values with statistical significance are in bold.

An increase was observed in the total population in reported respiratory alterations across all four categories over the study period, with increases in specific categories also noted in CF and PC (Table 3).

**Table 3:** Respiratory alterations according to categories by year, total population, and location. Bruminha Project, 2021, 2022, and 2023.

Variables	Total			Aranha			Córrego do Feijão			Parque da Cachoeira			Tejuco		
	2021	2022	2023	2021	2022	2023	2021	2022	2023	2021	2022	2023	2021	2022	2023
	Yes (%)	Yes (%)	Yes (%)	Yes (%)	Yes (%)	Yes (%)	Yes (%)	Yes (%)	Yes (%)	Yes (%)	Yes (%)	Yes (%)	Yes (%)	Yes (%)	Yes (%)
Respiratory allergy	32 (15.0)	53 (26.5)	28 (21.4)	8 (8.2)	27 (30.3)	16 (24.6)	4 (14.3)	7 (21.2)	2 (15.4)	14 (35.0)	10 (25.6)	7 (20.0)	6 (12.5)	9 (23.1)	3 (16.7)
p-value	-	**0.001**	**0.01**	-	**0.001**	**0.005**	-	0.50	0.95	-	0.50	0.25	-	0.25	0.75
Upper airway disorders^a^	60 (28.8)	64 (32.0)	66 (50.4)	25 (26.6)	29 (32.6)	29 (44.6)	6 (21.4)	8 (24.2)	7 (53.8)	12 (30.8)	10 (25.6)	22 (62.9)	17 (36.2)	17 (43.6)	8 (44.4)
p-value	-	**0.001**	**0.001**	-	0.25	0.75	-	0.9	**0.050**	-	0.75	**0.01**	-	0.50	0.75
Lower airway disorders^b^	36 (16.7)	42 (21.2)	35 (26.7)	17 (17.3)	16 (18.2)	15 (23.1)	1 (3.4)	7 (21.2)	5 (38.5)	9 (22.5)	10 (25.6)	9 (25.7)	9 (18.4)	9 (23.1)	6 (33.3)
p-value	-	0.25	**0.02**	-	0.9	0.50	-	**0.05**	**0.005**	-	0.75	0.75	-	0.75	0.25
Respiratory symptoms^c^	144 (66.7)	161 (80.5)	107 (81.7)	66 (67.3)	77 (86.5)	53 (81.5)	17 (58.6)	23 (69.7)	12 (92.3)	26 (65.0)	29 (74.4)	29 (82.9)	35 (71.4)	32 (82.1)	13 (72.2)
p-value	-	**0.001**	**0.005**	-	**0.005**	**0.05**	-	0.50	0.05	-	0.50	0.10	-	0.75	0.95

^a^ Rhinitis/sinusitis and otitis.

^b^ Pneumonia, asthma/wheezing, and bronchitis.

^c^ Cough, wheezing, shortness of breath, nasal congestion/runny nose, recurrent sneezing, rattling sounds/discharge, and ear pain.

p- value: values with statistical significance are highlighted.

### Metal Exposure Profile

The detection rate of arsenic in urine samples remained at 100% throughout the three years, both in the total population and across the four locations. In the total population, the percentage of samples exceeding the reference value (RV = 10 μg/gr of creatinine) increased from 42% in 2021 to 57% in 2023. In AR, the percentage of samples above the RV for arsenic remained in the range of 50%–52.3% over the three years. Conversely, in localities close to the disaster area (PC and CF) and in the area of ongoing mining activity (TJ), there was an increase in this percentage from 2021 (PC = 29%; CF = 29%; TJ = 37.5%) to 2023 (PC = 54%; CF = 62.5%; TJ = 72%).

All other metals analyzed showed an increase in detection rates in 2023 compared to 2022, reaching levels similar to those observed in 2021. In 2023, lead and mercury had a 100% detection rate in urine samples across all locations (except for mercury in PC, which was 96%). However, only three samples (2.5%) exceeded the RV in 2023 (PC – lead) (Table 4).

**Table 4:** Metal detection rate and proportion of samples above the reference value in the total population and by locality, according to year of study. Bruminha Project, 2021, 2022, and 2023.

Location	Metal	2021		2022		2023	
		Detection rate	% >RV	Detection rate	% >RV	Detection rate	% >RV
Total	As	172 (100)	72 (42.0)	131 (100)	58 (44.2)	88 (100)	50 (57)
	Cd	40 (23.2)	0	24 (18.3)	0	43 (48.8)	0
	Hb	110 (63.9)	2 (1.2)	52 (39.7)	1 (0.7)	88 (100)	0
	Mn	144 (83.7)	2 (1.2)	91 (69.5)	0	53 (60.2)	0
	Pb	153 (88.9)	23 (13)	64 (48.8)	0	88 (100)	6 (6.8)
		**Detection rate**	**% >RV**	**Detection rate**	**% >RV**	**Detection rate**	**% >RV**
Aranha	As	78 (100)	39 (50.0)	60 (100)	30 (50.0)	44 (100)	23 (52.3)
	Cd	19 (24.3)	0	9 (18.5)	0	24 (53.3)	0
	Hb	45 (57.7)	1 (1.3)	25 (41.7)	1 (1.6)	45 (100)	0
	Mn	63 (80.7)	1 (1.3)	43 (71.6)	0	20 (44.4)	0
	Pb	73 (93.6)	9 (11.5)	46 (77.0)	3 (5.0)	45 (100)	3 (6.8)
		**Detection rate**	**% >RV**	**Detection rate**	**% >RV**	**Detection rate**	**% >RV**
PC	As	31 (100)	9 (29)	24 (100)	15 (37.5)	24 (100)	13 (54.2)
	Cd	6 (19.3)	0	7 (29.2)	0	11 (45.8)	0
	Hb	21 (67.7)	1 (3.2)	6 (25.0)	0	23 (96)	0
	Mn	26 (83.8)	0	20 (83.3)	0	21 (87.5)	0
	Pb	24 (77.4)	2 (6.4)	7 (29.2)	0	24 (100)	3 (2.5)
		**Detection rate**	**% >RV**	**Detection rate**	**% >RV**	**Detection rate**	**% >RV**
CF	As	23 (100)	9 (29)	19 (100)	4 (21.1)	8 (100)	5 (62.5)
	Cd	3 (13.0)	0	1 (5.3)	0	2 (12.5)	0
	Hb	18 (78.2)	0	11 (57.9)	0	8 (100)	0
	Mn	20 (86.9)	0	4 (21.0)	0	2 (25.0)	0
	Pb	18 (78.2)	2 (8.7)	1 (5.3)	0	8 (100)	0
		**Detection rate**	**% >RV**	**Detection rate**	**% >RV**	**Detection rate**	**% >RV**
Tejuco	As	40 (100)	15 (37.5)	28 (100)	9 (32.2)	11 (100)	8 (72.2)
	Cd	12 (30.0)	0	7 (25.0)	0	7 (63.6)	0
	Hb	26 (65.0)	0	10 (35.7)	0	11 (100)	0
	Mn	35 (87.5)	1 (2.5)	24 (85.7)	0	10 (90.9)	0
	Pb	36 (90.0)	10 (25.0)	10 (35.7)	0	11 (100)	0

CF: Córrego do Feijão; PC: Parque da Cachoeira; RV: reference values for normality; Arsenic (As) = 10 μg/gr of creatinine; Cadmium (Cd) = 2 μg/gr of creatinine; Lead (Pb) = 1.7 μg/L of urine; Manganese (Mn) = 1 to 8 μg/L; Mercury (Hg) = 5 μg/gr of creatinine.

The analysis of the interquartile range (IQR) of metal concentrations showed an increase in the median urinary arsenic concentrations between 2021 (9.35 μg/g; IQR = 5.45–13.9) and 2023 (10.8 μg/g; IQR = 7.0–15.3) in the total number of children evaluated (p = 0.064). In PC, this increase was statistically significant (2021 = 6.3 μg/g; IQR = 3.4–13.2; 2023 = 10.4 μg/g; IQR = 6.02–16.35; p = 0.015). CF and TJ also showed increases in median arsenic concentrations and IQR values between 2021 and 2023, but without statistical significance (p > 0.10). In AR, the distribution of arsenic concentrations over the three years showed no statistical variation (p = 0.574) (Table 5).

**Table 5:** Distribution of urinary metal concentrations in the study population by location and year. Bruminha Project, 2021, 2022, and 2023.

Location	Metal	2021	2022	2023	p-value
		Median (IQR)	Median (IQR)	Median (IQR)	
Total	As (µg/g)	9.35 (5.45–13.9)	8.6 (5.1–13.3)	10.8 (7.0–15.3)	**0.064**
	Cd (µg/g)	<LOD	<LOD	<LOD (<LOD–0.1)	0.001
	Hg (µg/g)	0.30 (0.18–0.83)	<LOD (<LOD–0.7)	0.20 (0.1–0.3)	0.008
	Mn (µg/L)	0.35 (0.2–0.68)	0.4 (<LOD–0.8)	0.3 (0.3–0.9)	0.497
	Pb (μg/L)	0.8 (0.4–1.3)	<LOD (<LOD–0.5)	0.56 (0.3–0.9)	0.000
	**Metal**	**Median (IQR)**	**Median (IQR)**	**Median (IQR)**	
Aranha	As (µg/g)	10.40 (6.38–15.4)	9.8 (5.7–14.2)	10.70 (6.65–14.95)	0.574
	Cd (µg/g)	<LOD	<LOD	0.10 (<LOD–0.10)	0.003
	Hg (µg/g)	0.30 (0.20–0.55)	0.1 (<LOD–0.53)	0.20 (0.1–0.3)	0.413
	Mn (µg/L)	0.40 (0.2–0.7)	0.5 (<LOD–0.78)	<LOD	0.009
	Pb (μg/L)	0.70 (0.40–1.20)	0.5 (0.13–0.8)	0.45 (0.28–0.80)	**0.000**
	**Metal**	**Median (IQR)**	**Median (IQR)**	**Median (IQR)**	
Parque da Cachoeira	As (µg/g)	6.3 (3.4–13.2)	8.65 (5.4–16.2)	10.40 (6.02–16.35)	**0.015**
	Cd (µg/g)	<LOD	<LOD (<LOD–0.2)	<LOD (<LOD–0.10)	0.05
	Hg (µg/g)	1.30 (0.15–1.3)	<LOD (<LOD–0.25)	0.20 (0.10–0.30)	0.164
	Mn (µg/L)	0.20 (0.1–0.3)	0.45 (0.13–1.02)	0.55 (0.3–1.18)	**0.000**
	Pb (μg/L)	0.60 (0.33–0.98)	< LOD (<LOD–0.1)	0.85 (0.39–1.34)	0.000
	**Metal**	**Median (IQR)**	**Median (IQR)**	**Median (IQR)**	
Córrego do Feijão	As (µg/g)	8.60 (3.5–8.6)	5.4 (2.95–9.8)	11.30 (7.1–22.15)	0.112
	Cd (µg/g)	<LOD	<LOD	-	0.528
	Hg (µg/g)	0.2 (<LOD–0.83)	0.1 (<LOD–0.1)	0.18 (0.1– 0.25)	0.274
	Mn (µg/L)	0.5 (0.23–0.7)	<LOD	-	0.000
	Pb (μg/L)	0.75 (0.43–1.18)	<LOD	0.32 (0.145–0.67)	0.000
	**Metal**	**Median (IQR)**	**Median (IQR)**	**Median (IQR)**	
Tejuco	As (µg/g)	9.20 (5.48–12.7)	7.5 (4.1–11.1)	12.00 (6.9–13.0)	0.318
	Cd (µg/g)	<LOD	<LOD (<LOD–0.2)	0.10 (<LOD–0.1)	0.098
	Hg (µg/g)	0.2 (0.2–0.9)	<LOD (<LOD–0.2)	0.20 (0.1–0.3)	0.077
	Mn (µg/L)	0.4 (0.2–0.9)	0.7 (0.3–1.0)	0.60 (0.5–0.9)	0.435
	Pb (μg/L)	1.15 (0.1–1.6)	<LOD (<LOD–0.2)	0.61 (0.4–0.87)	0.000

p-value: values with statistical significance are highlighted.

As: arsenic; Cd: cadmium; Hg: mercury; Mn: manganese; Pb: Lead. LOD: limit of detection; IQ: interquartile range.

Despite the increase in urinary lead detection rates in 2023 (100%), there was no significant variation in the median or interquartile range of concentrations in total and across all locations—except AR, where a significant decrease (p = 0.000) was observed in median concentrations over the three years (2021 = 0.70 μg/L; 2022 = 0.50 μg/L; 2023 = 0.45 μg/L). Regarding mercury and cadmium, the increase in detection rates in 2023 did not change the medians of these metals in urine. A significant increase (p = 0.000) in the median and IQR for manganese was observed only in PC over the three years, although levels remained below the RV.

## DISCUSSION

The results of this study showed an increase in child exposure to the evaluated metal residues, with higher detection rates observed in 2023 compared to 2021. Arsenic was detected in 100% of urine samples across all three years of the project, while detection rates for lead, mercury, and cadmium increased from 88.9%, 63.9%, and 23.2% in 2021 to 100%, 100%, and 48.8% in 2023, respectively.

However, it is not yet possible to establish a trend of increasing exposure to higher concentrations of lead, mercury, and cadmium residues, due to the variation observed over the three-year period. In 2022, 25% of the samples analyzed—both in total and by location—were below the detection limit of the method, making it premature to establish a definitive trend. Over the three-year period, a significant decrease in median lead concentrations was observed at AR, while the remaining metals remained below the RV used.

Regarding arsenic concentrations, there was an increase in the total percentage of children with urinary concentrations above the RV over the three years (2021 = 42%; 2022 = 44%; 2023 = 57%), as well as in locations close to the disaster area (PC and CF) and the area of active mining (TJ) in 2023 compared to 2021. Across all three years, the medians and the distribution range of arsenic concentrations increased in the total population (p = 0.064), and significantly so among children living in PC (p = 0.015), suggesting a trend towards greater exposure to this metal.

Overall, the results suggest a broader environmental dissemination of these residues and, consequently, a greater number of exposed children. This may be due to the resumption of mining activity in the region, especially from 2022 onward, which was interrupted after the disaster and during the covid-19 pandemic. This greater dissemination of waste may be associated with increased reports of respiratory alterations.

Before the disaster, the main sources of drinking water reported across all locations were shared cisterns and wells or springs, reflecting socio-environmental vulnerability^13^. After the disaster, the mining company responsible began supplying mineral water to residents of the affected areas—including TJ, a community located very close to and at a lower elevation than a large mining area that is now active again. PC and CF are located within the perimeter of ongoing remediation activities. In addition to the potential contamination of groundwater, it is important to highlight the possibility of inhalation of dust generated by these activities.

The term “background exposure” refers to the concentrations of chemical substances to which the general population is exposed, based on the presence of these substances in the environment (air, water, soil, food, dust, etc.)^14^. Several countries have conducted studies to establish background levels of environmental exposure to metals in their populations^15^. From 2017 to 2018, the National Report on Human Exposure to Environmental Chemicals, part of the U.S. National Health and Nutrition Survey^18^, reported the following values for children aged three to five: a median urinary arsenic concentration of 8.19 μg/g (7.54–8.95) and a 95th percentile of 42.4 μg/g (27.8–70.6); a median urinary lead concentration of 0.481 μg/g (0.444–0.538) and a 95th percentile of 1.65 μg/g (1.24–2.31); and a 95th percentile for urinary mercury of 0.390 μg/g (0.210–1.19), with a median below the detection limit. In the Bruminha Project, urinary arsenic medians over the three years were higher than those reported in the U.S. (2021 = 9.35 μg/g; 2022 = 8.6 μg/g; 2023 = 10.85 μg/g), whereas the 95th percentiles were lower (2021 = 27.01 μg/g; 2022 = 24.6 μg/g; 2023 = 25.66 μg/g). Both the medians and 95th percentiles for urinary lead and mercury concentrations were higher than U.S. values, except in 2022.

However, comparisons between different populations may be prone to error due to variability in economic, social, and cultural characteristics; dietary patterns; environmental interactions and exposure; existing production processes (industries, etc.); and specific environmental characteristics (geological, climatic, hydrological, urbanization, etc.) of a given location, all of which can lead to different levels of population exposure. Another factor that complicates such comparisons is the time period in which the study was conducted. Exposure to environmental chemicals changes over time, and variations in exposure patterns can be observed within the same population across different years.

A significant reduction in the risk of neurodevelopmental delay was observed in the study population over the three years. Children’s improved performance in tasks related to language, cognitive, and motor skill development may be attributed to the return of school activities and children’s social lives following the isolation caused by the covid-19 pandemic^19^. However, it is important to note that this improvement was significantly greater among children living in communities impacted by the disaster. Even before the isolation caused by the pandemic, children in these locations were experiencing social disruption in their family, school, and community environments. Sudden life changes, emotional distress, and a sense of disorder and insecurity—both personal and collective—had already formed the social backdrop for these families during the pre-pandemic period. It is plausible to assume that the progressive return to social normalcy after 2022 contributed to a more expressive recovery among these children, although the increase in maternal education and household income over the years may also be contributing factors.

The end of social distancing measures may also be associated with the improvement observed in anthropometric assessments in the study population, as children regained the opportunity to play outdoors and engage in sport activities.

A limitation of this study is the assessment of respiratory alterations based on reports from parents or caregivers, without validation using medical records. Likewise, the Denver test is a screening tool, and its results indicate a suspected developmental delay that should be confirmed by means of an accurate clinical evaluation.

It is important to emphasize that, over the three years, no associations were observed between exposure to mining residues and any of the studied health outcomes^20^. The Bruminha Project does not aim to establish causal relationships. In cases of long-term exposure to low doses, as in this study, the toxic effects of the substances identified may manifest in broad and delayed ways, potentially triggering or aggravating morbid processes with multifactorial etiologies.

For this reason, it is essential that SUS also structures its guidelines and actions according to specificities of the productive processes in each territory. In regions such as the Iron Quadrangle of Minas Gerais, where mining is the predominant economic activity —and one that inherently generates environmental residues and, consequently, exposure risks for the surrounding population—it is essential to implement environmental health surveillance. This should involve coordinated efforts between municipal and state health and environment departments, aiming to evaluate human exposure to such residues. Likewise, primary care health teams—responsible for coordinating care for the assigned population—must be trained on the potential toxic effects of these residues on human health, the identification of vulnerable population groups, and the implementation of protocols for monitoring, investigation, and care. This includes the possibility of requesting specific toxicological tests, as well as the organization of the healthcare network with multidisciplinary teams at the primary level (formerly known as Expanded Family Health Support Centers in Brazil), and at the secondary and tertiary levels of care, involving specialists and institutions with expertise in the field.

The findings described above reveal a scenario of ongoing exposure among the Bruminha Project study population to metal residues that are potentially toxic to children’s health, with no indication of associated health effects at the time of the study. Every illness process results not only from exposure to singular chemical, physical, or biological agents, but also from a broader framework of economic, social, cultural, genetic, and environmental conditions that structure individuals’ perception and experience of health. In the case of children, factors such as nutritional status, developmental stage, metabolic and immune capacity, and the overall maturity of the organism are determining factors in the relationship between health and disease.

This scenario highlights the critical need for coordination between primary healthcare teams and managers and the municipal, state, and federal environmental and health surveillance sectors, aiming to build a SUS that meets the specific productive profiles of the territories it serves.

## Data Availability

As the cohort study is still in development, the data are not yet available.
