# Supplementary material for: Health profile and metal exposure over three years in a child cohort study, Bruminha Project
Source: Rev Saude Publica. 2025 Nov 17;59:e30. doi: 10.11606/s1518-8787.2025059006931 (PMC12666785; doi:10.11606/s1518-8787.2025059006931)
Supplement: Translation

# Translation: Perfil de saúde e exposição a metais em três anos de uma coorte infantil, Projeto Bruminha

**Authors:** Carmen Ildes Rodrigues Fróes Asmus, Maíra Lopes Mazoto, Nataly Damasceno de Figueiredo, Herling Gregorio Aguilar Alonzo, Renan Duarte dos Santos Saraiva, Aline de Souza Espindola Santos, Ana Paula Natividade de Oliveira, Ivisson Carneiro Medeiros da Silva, Michele Alves Costa, Leiliane Coelho André, Sérgio Viana Peixoto, Volney de Magalhães Câmara

**Affiliations:** I Brasil Universidade Federal do Rio de Janeiro. Instituto de Estudos de Saúde Coletiva. Rio de Janeiro, RJ, Brasil; II Brasil Universidade Estadual de Campinas. Faculdade de Ciências Médicas. Campinas, SP, Brasil; III Brasil Fundação Oswaldo Cruz. Escola Nacional de Saúde Pública. Rio de Janeiro, RJ, Brasil; IV Brasil Universidade Federal de Minas Gerais. Faculdade de Farmácia. Belo Horizonte, MG, Brasil; V Brasil Fundação Oswaldo Cruz. Instituto René Rachou. Belo Horizonte, MG, Brasil

**Keywords:** Efeitos de Desastres na Saúde, Estudos de Coorte, Metais, Saúde Infantil

## Abstract

**OBJETIVO:**

Descrever o perfil de saúde e o padrão de exposição aos resíduos de minério nos três anos de uma coorte com crianças residentes nas localidades atingidas pelo desastre de rompimento da barragem de mineração em Brumadinho (MG), em 2019.

**MÉTODOS:**

Coorte prospectiva cuja população de estudo é formada por todas as crianças entre 0 e 6 anos residentes em quatro localidades selecionadas, sendo três (Parque da Cachoeira, Córrego do Feijão e Tejuco) na área do desastre (área exposta) e uma (Aranha) situada a 10 km de distância (área não exposta). Foram realizadas avaliações do crescimento pôndero-estatural, desenvolvimento neuromotor e cognitivo e alterações respiratórias, bem como coletadas amostras de urina para análise de chumbo, arsênio, cádmio, mercúrio e manganês pelo método de espectrometria de massas com plasma indutivamente acoplado (ICP-MS).

**RESULTADOS:**

O percentual médio de crianças avaliadas foi de 80% da população prevista no período. Houve aumento do percentual de crianças detectadas com níveis de arsênio urinário acima do valor de referência ao longo dos três anos (2021 = 42%; 2022 = 44%; 2023 = 57%) e das taxas de detecção de chumbo (88,9%) e mercúrio (63,9%) em 2021, para 100% em 2023. Houve aumento da mediana das concentrações de arsênio entre os anos de 2021 (9,35 µg/g; IQI = 5,45–13,9) e 2023 (10,8 µg/g; IQI = 7,0–15,3) no total de crianças avaliadas (p = 0,064), e de forma significativa nas crianças residentes na área exposta (p = 0,015, PC). Nos três anos ocorreu uma diminuição no percentual de alterações do neurodesenvolvimento e de sobrepeso e obesidade, e um aumento dos relatos de alterações respiratórias na população total, sem relação com a exposição a metais.

**CONCLUSÃO:**

Observa-se um cenário de exposição permanente da população de estudo aos metais analisados, mas ainda sem indicação de alterações de saúde associadas aos mesmos. É importante a organização do Sistema Único de Saúde de acordo com as especificidades dos processos produtivos de cada território.

## INTRODUÇÃO

Em 25 de janeiro de 2019, ocorreu o rompimento da Barragem I de rejeitos da Mina de Córrego do Feijão, na cidade de Brumadinho, estado de Minas Gerais, Brasil. O deslizamento de 12 milhões/m^3^ de lama, causando pelo menos 272 óbitos, atingiu 18 municípios, comprometeu a viabilidade e o fornecimento de água da Bacia do Rio Paraopeba e contaminou o solo e a produção de alimentos^1^.

Em junho de 2019, o Ministério da Saúde do Brasil propôs a realização de um Programa de Ações Integradas em Saúde em Brumadinho, com o objetivo de monitorar a população exposta aos impactos deste desastre, identificando as lacunas do conhecimento relativas aos efeitos a médio e longo prazo sobre a saúde, e propondo ações que instrumentalizassem o Sistema Único de Saúde (SUS) do país para o enfrentamento destes^2^. Esse Programa consiste em dois estudos longitudinais que foram iniciados no ano de 2021, o Projeto Saúde Brumadinho, coordenado pela Fundação Oswaldo Cruz de Minas Gerais e o Projeto Bruminha, coordenado pela Universidade Federal do Rio de Janeiro (UFRJ) com avaliações anuais da população, previstas para o período de cinco anos^2^.

O “Projeto Bruminha – Estudo Longitudinal da Saúde Infantil em Brumadinho” é uma coorte prospectiva composta por crianças entre 0 e 6 anos de idade, residentes em quatro localidades selecionadas. Ele investiga os impactos do desastre sobre a saúde infantil, avaliando a ocorrência de alterações antropométricas e do desenvolvimento neurológico, e afecções respiratórias, além do perfil de exposição aos resíduos de metais. Este artigo apresenta o perfil de saúde e de exposição aos resíduos ao longo dos três anos iniciais de realização da coorte.

## MÉTODOS

O protocolo do Projeto Bruminha está descrito em Asmus et al.^3^

### Área de Estudo

A área de estudo do Projeto Bruminha é composta por quatro localidades. Córrego do Feijão (CF) e Parque da Cachoeira (PC) foram diretamente afetadas, com residências próximas (1,5 km) a lama dos rejeitos de mineração. A localidade de Tejuco (TJ) foi incluída devido a sua posição geográfica, em nível abaixo de uma área de mineração. A localidade de Aranha (AR) situa-se a uma distância superior a 10 km da lama de rejeitos, e foi considerada fora da área do desastre.

### População de Estudo

Todas as crianças residentes nas localidades estudadas até a idade limite de 6 anos e 11 meses nos anos de 2021, 2022 e 2023 foram convidadas a participar do Projeto. Considerando-se a situação de interesse, na qual podem ocorrer mudanças na condição de exposição, a coorte se estruturou com uma população dinâmica, com entrada e/ou saída de participantes ao longo do período de seguimento. A listagem do total de crianças da população de estudo prevista foi obtida por meio dos registros de residentes locais constantes na Secretaria de Saúde do Município.

### Instrumentos, Coleta de Dados e Coleta de Amostras de Urina

1. Questionário socioambiental: renda *per capita*; idade e escolaridade maternas; raça/cor, sexo e idade da criança; saneamento; e fonte de água potável.
2. Avaliações antropométricas: peso e comprimento/altura^4^.
3. Formulário clínico: recordatório dos pais sobre afecções respiratórias das crianças – tosse, sibilo, dificuldade para respirar, congestão nasal/coriza, roncos/secreção, espirros recorrentes e otalgia (recordatório de 15 dias), pneumonia, asma/sibilância ou sibilo, bronquite, rinite/sinusite, alergia respiratória e otite (recordatório de 12 meses).
4. Teste de Triagem de Desenvolvimento – Denver II. Esse teste é um instrumento de triagem constituído por um conjunto de 125 itens representativos de habilidades em quatro áreas do desenvolvimento (pessoal-social, motora fino-adaptativa, linguagem e motora grossa). Ele é aplicado por meio da realização de tarefas pela criança e por relato dos seus cuidadores/pais, para verificação de suas habilidades nessas quatro áreas. De acordo com o desempenho da criança, registra-se: a) “Passou” quando consegue realizar a tarefa adequadamente; b) “Falhou” quando não consegue realizar a tarefa; c) “Sem oportunidade” quando não é uma atividade comum ao seu cotidiano; ou d) “Recusa” quando se nega a realizar a tarefa proposta. Quando há falha em uma habilidade que 75% a 90% das crianças da mesma idade conseguem executar, a tarefa é codificada como “Cautela”. Quando há falha em atividades em que 90% ou mais das crianças da mesma idade conseguem realizar, codifica-se a tarefa como “Atraso”^5^.
5. Amostras de urina das crianças: para dosagem das concentrações dos metais arsênio, cádmio, chumbo, mercúrio^6^ e manganês^7^.

Os valores de referência (VR) para as concentrações urinárias de arsênio, cádmio e mercúrio foram baseados nos valores estabelecidos pela Norma Reguladora número 7 (NR–7, 1994) do Ministério do Trabalho, cuja versão em vigor no primeiro ano de realização do estudo (julho de 2021) previa um VR para populações não-expostas ocupacionalmente a esses metais^8^. Para o manganês e chumbo urinários foram utilizadas as referências da Agency for Toxic Substances and Disease Registry (ATSDR)^9^ e Saravanabhavan et al.^10^

### Análise Laboratorial

As concentrações de metais foram analisadas pelo método de espectrometria de massas com plasma indutivamente acoplado (ICP-MS). O limite de detecção e o limite de quantificação para arsênio (total), cádmio, chumbo, mercúrio e manganês foram de 0,1 μg l-1^11^.

### Análise Estatística

As características sociodemográficas da população foram descritas para o total da população estudada e por localidade. Para as variáveis categóricas, foram calculadas as frequências relativas e para as medidas contínuas, medidas de tendência central e de variabilidade.

A descrição do perfil antropométrico, neurodesenvolvimento e sintomas respiratórios foi feita para a população total e para as localidades segundo ano de avaliação. As medidas antropométricas foram categorizadas conforme o Índice de Massa Corpórea (IMC) para essa faixa etária^4^: baixo peso (< 18,5 kg/altura^2^); eutróficos (18,6 a 24,9 kg/altura^2^); e sobrepeso/obeso (> 30 kg/altura^2^). As afecções respiratórias foram organizadas em: vias aéreas superiores (rinite/sinusite e otite); vias aéreas inferiores (pneumonia, asma/sibilância/sibilo e bronquite); e sinais e sintomas respiratórios (tosse, sibilo, dificuldade para respirar, congestão nasal/coriza, espirros recorrentes, roncos/secreções e otalgia). A alergia respiratória foi avaliada separadamente^12^. A avaliação do neurodesenvolvimento foi categorizada em dois grupos: 1) Denver–normal: crianças que realizaram todas as tarefas e crianças que apresentaram “cautela” em apenas um dos domínios; 2) Denver–risco: crianças que apresentaram “cautelas” em mais de um domínio, ou “atrasos” em um, dois, três ou nos quatro domínios. A ocorrência de diferenças estatísticas foi verificada por meio do teste qui-quadrado (teste de correção de continuidade de Yate e Pearson).

Para análise do padrão de exposição aos metais calculou-se a taxa de detecção para cada metal, definida como o percentual de amostras de urina nas quais o metal foi detectado, em relação ao total de amostras válidas de urina analisadas. Foram consideradas válidas as amostras urinárias coletadas com concentração de creatinina entre 0,3g/L e 3,3 g/L.

A mediana e o intervalo interquartílico (P25–P75) das concentrações dos metais na urina foram estabelecidos para cada localidade e ano de avaliação. Para comparação entre os anos em cada localidade foi aplicado o teste Kruskal Wallis de amostras independentes, para verificar a existência de diferença na variabilidade das concentrações de cada metal.

### Aspectos Éticos

O estudo foi aprovado pelo Comitê de Ética em Pesquisa da UFRJ, sob o parecer número 5.201.083.

## RESULTADOS

### Características da População de Estudo

O percentual médio de crianças avaliadas foi de 80% da população prevista ao longo dos três anos da pesquisa. Nos anos de 2021, 2022 e 2023, 42,9%, 33,8% e 53,4% tinham acima de quatro anos, respectivamente. Há maior prevalência do sexo masculino e de população não-branca (referência materna), exceto em 2022 (Tabela 1).

**Tabela 1:** Proporção de crianças avaliadas por localidade e características sociodemográficas. Projeto Bruminha, 2021, 2022 e 2023.

Variáveis	Total			Aranha			Córrego do Feijão			Parque da Cachoeira			Tejuco		
	2021	2022	2023	2021	2022	2023	2021	2022	2023	2021	2022	2023	2021	2022	2023
N. avaliado^a^	217 (62)	198 (105)	131 (74)	98 (60)	87 (104)	65 (77)	30 (59)	33 (91)	13 (88)	40 (69)	39 (108)	35 (106)	49 (65)	39 (100)	18 (49)
Idade - n (%)															
0–11 meses	29 (13,3)	41 (20,7)	8 (6,1)	10 (10,2)	11 (12,6)	2 (3,1)	5 (16,6)	9 (27,3)	1 (7,7)	6 (15)	11 (28,2)	3 (8,6)	8 (16,3)	10 (25,6)	2 (11,1)
1–2 anos	28 (12,9)	33 (16,7)	21 (16,0)	16 (16,3)	22 (25,3)	8 (12,3)	3 (10)	3 (9,1)	2 (15,4)	5 (12,5)	3 (7,7)	9 (25,7)	4 (8,2)	5 (12,8)	2 (11,1)
> 2–4 anos	67 (30,9)	57 (28,8)	32 (24,4)	28 (28,6)	25 (28,7)	16 (24,6)	11 (36,7)	9 (27,3)	4 (30,7)	15 (37,5)	13 (33,4)	7 (20,0)	13 (26,5)	10 (25,6)	5 (27,8)
> 4 anos	93 (42,9)	67 (33,8)	70 (53,4)	44 (44,9)	29 (33,4)	39 (60,0)	11 (36,7)	12 (36,3)	6 (46,2)	14 (35,0)	12 (30,7)	16 (45,7)	24 (49,0)	14 (36,0)	9 (50,0)
N. total	217	198	131	98	87	65	30	33	13	40	39	35	49	39	18
Sexo - n (%)															
Feminino	103 (47,5)	99 (50,0)	55 (42,0)	44 (44,9)	46 (52,9)	30 (46,1)	13 (43,3)	17 (51,5)	5 (38,5)	19 (47,5)	16 (41,0)	13 (37,2)	27 (55,1)	20 (51,3)	7 (38,9)
Masculino	114 (52,5)	99 (50,0)	76 (58,0)	54 (55,1)	41 (47,1)	35 (53,9)	17 (56,7)	16 (44,5)	8 (61,5)	21 (52,5)	23 (59,0)	22 (62,8)	22 (44,9)	19 (48,7)	11 (61,1)
Total	217	198	131	98	87	65	30	33	13	40	39	35	49	39	18
Cor/raça - n (%)															
Branca	78 (38,8)	99 (50,0)	47 (36,4)	44 (48,3)	46 (52,9)	29 (45,3)	7 (26,9)	17 (51,5)	3 (27,2)	14 (37,8)	16 (41,0)	8 (22,9)	13 (27,7)	20 (51,3)	7 (38,8)
Não branca	123 (61,2)	99 (50,0)	82 (63,6)	47 (51,7)	41 (47,1)	35 (54,7)	19 (73,1)	16 (48,5)	9 (81,8)	23 (62,2)	23 (59,0)	27 (77,1)	34 (72,3)	19 (48,7)	11 (61,1)
Total^a^	201	198	129	91	87	64	26	33	11	37	39	35	47	39	18
Idade média da mãe															
Média (DP)	33 (9)	32 (6)	33 (6)	33 (9)	33 (6)	33 (6)	32 (9)	31 (5)	32 (6)	32 (9)	33 (6)	33 (5)	33 (10)	33 (8)	34,8 (6)
Escolaridade da mãe em anos de estudo															
1 a 9 anos	58 (33,2)	36 (27,7)	23 (20,4)	30 (37,0)	16 (27,0)	11 (20,0)	3 (14,3)	4 (21,0)	2 (18,2)	12 (33,3)	7 (25,0)	7 (22,6)	13 (35,1)	9 (38,0)	3 (18,7)
> 9 anos	117 (66,8)	94 (72,3)	90 (79,6)	51 (63,0)	43 (73,0)	44 (80,0)	18 (85,7)	15 (79,0)	9 (81,8)	24 (66,7)	21 (75,0)	24 (77,4)	24 (64,9)	15 (63,0)	13 (81,3)
Total	175	130	113	81	59	55	21	19	11	36	28	31	37	24	16
Esgotamento sanitário															
Fossa	175 (87,0)	152 (96,8)	113 (89,0)	87 (93,6)	70 (97,3)	55 (88,7)	25 (89,2)	24 (100)	12 (92,3)	36 (94,8)	31 (100)	34 (97,2)	27 (64,3)	27 (90,0)	12 (70,6)
Rede	8 (4,0)	5 (3,2)	10 (7,9)	3 (3,2)	2 (2,7)	7 (11,3)	2 (7,2)	0	1 (7,7)	1 (2,6)	0	0	2 (4,7)	3 (10)	2 (11,8)
Céu aberto/ rio/lago	18 (9,0)	0	4 (3,1)	3 (3,2)	0	0	1 (3,6)	0	0	1 (2,6)	0	1 (2,8)	13 (31,0)	0	3 (17,6)
Total	201	157	127	93	72	62	28	24	13	38	31	35	42	30	17
Fonte de água para consumo															
Mineral	125 (60,1)	100 (58,5)	83 (64,3)	24 (25,3)	17 (23,0)	26 (41,9)	28 (100)	25 (92,6)	11 (78,6)	37 (92,5)	32 (94,1)	34 (97,2)	36 (80,0)	26 (72,2)	12 (66,7)
Outras^b^	83 (39,9)	71 (41,5)	46 (35,7)	71 (74,7)	57 (77,0)	36 (58,1)	0	2 (7,4)	3 (21,4)	3 (7,5)	2 (5,9)	1 (2,8)	9 (20,0)	10 (27,8)	6 (33,3)
Total	208	171	129	95	74	62	28	27	14	40	34	35	45	36	18

DP: desvio padrão.

^a^ Número de crianças avaliadas e percentual em relação ao total de crianças esperadas de acordo com o cadastro da unidade de saúde local.

^b^ Outras fontes de água potável: cisterna coletiva e poço ou nascente.

A média de idade das mães/responsáveis em cada ano variou entre 32,4 (6,1) e 33 (9,0) anos. Observou-se um aumento do percentual de mães/responsáveis com mais de nove anos de estudo, no total (66,8%–79,6%) e por localidade, ao longo dos três anos da pesquisa. A fossa séptica foi o tipo de esgotamento sanitário mais prevalente em todos os anos de avaliação, no total de domicílios e por localidade. TJ apresentou o maior percentual de esgotamento sanitário do tipo céu aberto/rio/lago nos anos de 2021 (31%) e 2023 (17,6%). Em todos os três anos da pesquisa, a principal fonte de água para consumo humano, no total de domicílios avaliados, e nas localidades de PC, CF e TJ, foi água mineral. Em AR, o maior número de domicílios utilizou outras fontes de consumo de água (cisterna coletiva, poço ou nascente), que não a mineral, em todos os anos de estudo (Tabela 1).

### Perfil de Saúde

A maioria das crianças apresentou IMC normal em 2021 (76%), 2022 (83%) e 2023 (91%) com diminuição dos percentuais de sobrepeso e obesidade ao longo deste período, em todas as localidades estudadas.

Ao longo dos três anos de avaliação, houve uma redução significativa no percentual de crianças com resultado do teste de Denver II com risco de atraso, tomando-se o ano 2021 como referência. Na população total, a redução foi de aproximadamente 34% (p < 0,05) tanto para o ano de 2022, quanto para o ano de 2023. Quando analisado por localidade, apenas AR não apresentou diferença significativa em ambos os períodos analisados. Em CF (p = 0,005) e em PC (p = 0,040), a ocorrência de risco de atraso na avaliação do neurodesenvolvimento diminuiu em 97% e 48%, respectivamente, no ano de 2022, e na localidade de TJ (p = 0,01) em 66% no ano de 2023, em relação ao ano de 2021 (Tabela 2).

**Tabela 2:** Avaliação do neurodesenvolvimento e classificação do Índice de Massa Corporal por ano, população total e segundo localidade. Projeto Bruminha, 2021, 2022 e 2023.

Localidade	Neurodesenvolvimento n (%)		p^a^	Classificação de acordo com o IMC n (%)			p^a^
	Normal	Risco de atraso		Baixo peso	Normal	Sobrepeso/obeso	
Total							
2021	111 (57,5)	82 (42,5)	-	0	164 (76,0)	52 (24,0)	-
2022	137 (71,7)	54 (28,3)	**0,003**	3 (1,5)	165 (83,3)	30 (15,2)	**0,025**
2023	86 (71,7)	34 (28,3)	**0,010**	0	119 (90,8)	12 (9,2)	**0,001**
Aranha							
2021	59 (67,8)	28 (32,2)	-	0	72 (74,2)	25 (25,8)	-
2022	59 (68,6)	27 (31,4)	0,95	1 (1,1)	76 (84,4)	13 (15,6)	0,10
2023	40 (71,4)	16 (28,6)	0,64	0	56 (89,0)	7 (11,0)	**0,03**
Córrego do Feijão							
2021	12 (44,4)	15 (55,6)	-	0	25 (83,3)	5 (16,7)	-
2022	25 (83,3)	5 (16,7)	**0,005**	1 (3,3)	26 (83,9)	2 (6,4)	0,50
2023	10 (66,7)	5 (33,3)	0,16	0	13 (86,7)	2 (13,3)	0,90
Parque da Cachoeira							
2021	18 (50)	18 (50)	-	0	23 (57,5)	17 (42,5)	-
2022	28 (73,7)	10 (26,3)	**0,040**	1 (2,6)	31 (81,6)	6 (15,8)	**0,025**
2023	21 (67,7)	10 (32,3)	0,14	0	33 (94,3)	2 (5,7)	**0,001**
Tejuco							
2021	22 (51,2)	21 (48,3)	-	0	44 (89,8)	5 (10,2)	-
2022	25 (18,3)	12 (22,2)	0,13	0	32 (82,0)	7 (18,0)	0,50
2023	15 (83,3)	3 (16,7)	**0,01**	0	17 (94,4)	1 (5,6)	0,75

IMC: Índice de Massa Corporal.

^a^ Teste qui-quadrado para comparação das proporções observadas a cada ano, tomando-se 2021 como referência. Em negrito os valores que apresentam significância estatística.

Houve um aumento na população total dos relatos de alterações respiratórias nas quatro categorias estudadas ao longo dos anos, e para algumas categorias específicas em CF e PC (Tabela 3).

**Tabela 3:** Alterações respiratórias segundo categorias por ano, na população total e segundo localidade. Projeto Bruminha, 2021, 2022 e 2023.

Variáveis	Total			Aranha				Córrego do Feijão			Parque da Cachoeira			Tejuco		
	2021	2022	2023	2021		2022	2023	2021	2022	2023	2021	2022	2029	2021	2022	2023
	Sim (%)	Sim (%)	Sim (%)	Sim (%)		Sim (%)	Sim (%)	Sim (%)	Sim (%)	Sim (%)	Sim (%)	Sim (%)	Sim (%)	Sim (%)	Sim (%)	Sim (%)
Alergia respiratória	32 (15,0)	53 (26,5)	28 (21,4)		8 (8,2)	27 (30,3)	16 (24,6)	4 (14,3)	7 (21,2)	2 (15,4)	14 (35,0)	10 (25,6)	7 (20,0)	6 (12,5)	9 (23,1)	3 (16,7)
P-valor	-	0,001	0,01		-	0,001	0,005	-	0,50	0,95	-	0,50	0,25	-	0,25	0,75
Afecções de vias aéreas superiores^a^	60 (28,8)	64 (32,0)	66 (50,4)		25 (26,6)	29 (32,6)	29 (44,6)	6 (21,4)	8 (24,2)	7 (53,8)	12 (30,8)	10 (25,6)	22 (62,9)	17 (36,2)	17 (43,6)	8 (44,4)
P-valor	-	0,001	0,001		-	0,25	0,75	-	0,90	0,050	-	0,75	0,01	-	0,50	0,75
Afecções de vias aéreas inferiores^b^	36 (16,7)	42 (21,2)	35 (26,7)		17 (17,3)	16 (18,2)	15 (23,1)	1 (3,4)	7 (21,2)	5 (38,5)	9 (22,5)	10 (25,6)	9 (25,7)	9 (18,4)	9 (23,1)	6 (33,3)
P-valor	-	0,25	0,02		-	0,90	0,50	-	0,05	0,005	-	0,75	0,75	-	0,75	0,25
Sinais e sintomas respiratórios^c^	144 (66,7)	161 (80,5)	107 (81,7)		66 (67,3)	77 (86,5)	53 (81,5)	17 (58,6)	23 (69,7)	12 (92,3)	26 (65,0)	29 (74,4)	29 (82,9)	35 (71,4)	32 (82,1)	13 (72,2)
P-valor	-	**0,001**	**0,005**		-	**0,005**	**0,05**	-	0,50	0,05	-	0,50	0,10	-	0,75	0,95

^a^ Rinite/sinusite e otite.

^b^ Pneumonia, asma/sibilância/sibilo e bronquite.

^c^ Tosse, sibilo, dificuldade para respirar, congestão nasal/coriza, espirros recorrentes, roncos/secreções e otalgia.

P-valor: destacados em negrito os valores com significância estatística.

### Perfil de Exposição aos Metais

A taxa de detecção do arsênio nas urinas analisadas permaneceu em 100%, ao longo dos três anos, na população total e nas quatro localidades estudadas. Na população total, o percentual de amostras acima do VR (VR = 10 µg/gr de creatinina) passou de 42% em 2021 para 57% em 2023. Na localidade de AR, o percentual de amostras acima do VR para arsênio manteve-se na faixa de 50%–52,3%, ao longo dos três anos, enquanto nas localidades situadas próximas a área do desastre (PC e CF) e de mineração ativa (TJ) houve um aumento desse percentual de 2021(PC = 29%; CF = 29%; TJ = 37,5%) para 2023 (PC = 54%; CF = 62,5%; TJ = 72%).

Todos os outros metais analisados apresentaram um aumento da taxa de detecção no ano de 2023 em relação ao ano de 2022, com percentuais semelhantes aos do ano de 2021. Chumbo e mercúrio apresentaram uma taxa de detecção na urina de 100% em todas as localidades no ano de 2023 (PC–mercúrio = 96%). No entanto, apenas em três (2,5%) amostras analisadas o VR é ultrapassado em 2023 (PC-chumbo) (Tabela 4).

**Tabela 4:** Taxa de detecção dos metais e proporção de amostras acima do valor de referência na população total e por localidade, segundo ano de estudo. Projeto Bruminha, 2021, 2022 e 2023.

Local	Metal	2021		2022		2023	
		Taxa de detecção	% >VR	Taxa de detecção	% >VR	Taxa de detecção	% >VR
Total	As	172 (100)	72 (42,0)	131 (100)	58 (44,2)	88 (100)	50 (57)
	Cd	40 (23,2)	0	24 (18,3)	0	43 (48,8)	0
	Hg	110 (63,9)	2 (1,2)	52 (39,7)	1 (0,7)	88 (100)	0
	Mn	144 (83,7)	2 (1,2)	91 (69,5)	0	53 (60,2)	0
	Pb	153 (88,9)	23 (13)	64 (48,8)	0	88 (100)	6 (6,8)
		**Taxa de detecção**	**% >VR**	**Taxa de detecção**	**% >VR**	**Taxa de detecção**	**% >VR**
Aranha	As	78 (100)	39 (50,0)	60 (100)	30 (50,0)	44 (100)	23 (52,3)
	Cd	19(24,3)	0	9 (18,5)	0	24 (53,3)	0
	Hg	45 (57,7)	1 (1,3)	25 (41,7)	1 (1,6)	45 (100)	0
	Mn	63 (80,7)	1 (1,3)	43 (71,6)	0	20 (44,4)	0
	Pb	73 (93,6)	9 (11,5)	46 (77,0)	3 (5,0)	45 (100)	3 (6,8)
		**Taxa de detecção**	**% >VR**	**Taxa de detecção**	**% >VR**	**Taxa de detecção**	**% >VR**
PC	As	31 (100)	9 (29)	24 (100)	15 (37,5)	24 (100)	13 (54,2)
	Cd	6 (19,3)	0	7 (29,2)	0	11 (45,8)	0
	Hg	21 (67,7)	1 (3,2)	6 (25,0)	0	23 (96)	0
	Mn	26 (83,8)	0	20 (83,3)	0	21 (87,5)	0
	Pb	24 (77,4)	2 (6,4)	7 (29,2)	0	24 (100)	3 (2,5)
		**Taxa de detecção**	**% >VR**	**Taxa de detecção**	**% >VR**	**Taxa de detecção**	**% >VR**
CF	As	23 (100)	9 (29)	19 (100)	4 (21,1)	8 (100)	5 (62,5)
	Cd	3 (13,0)	0	1 (5,3)	0	2 (12,5)	0
	Hg	18 (78,2)	0	11 (57,9)	0	8 (100)	0
	Mn	20 (86,9)	0	4 (21,0)	0	2 (25,0)	0
	Pb	18 (78,2)	2 (8,7)	1 (5,3)	0	8 (100)	0
		**Taxa de detecção**	**% >VR**	**Taxa de detecção**	**% >VR**	**Taxa de detecção**	**% >VR**
Tejuco	As	40 (100)	15 (37,5)	28 (100)	9 (32,2)	11 (100)	8 (72,2)
	Cd	12 (30,0)	0	7 (25,0)	0	7 (63,6)	0
	Hg	26 (65,0)	0	10 (35,7)	0	11 (100)	0
	Mn	35 (87,5)	1 (2,5)	24 (85,7)	0	10 (90,9)	0
	Pb	36 (90,0)	10 (25,0)	10 (35,7)	0	11 (100)	0

CF: Córrego do Feijão; PC: Parque da Cachoeira; VR: valores de referência da normalidade; Arsênio (As) = 10 µg/gr de creatinina; Cádmio (Cd) = 2 µg/gr de creatinina; Chumbo (Pb) = 1,7 µg/L de urina; Manganês (Mn) = 1 a 8 μg/L; Mercúrio (Hg) = 5 µg/gr de creatinina.

Na análise do intervalo de distribuição das concentrações dos metais (IQI), observa-se um aumento da mediana das concentrações de arsênio entre os anos de 2021 (9,35 µg/g; IQI = 5,45–13,9) e 2023 (10,8 µg/g; IQI = 7,0–15,3) no total de crianças avaliadas (p = 0,064). Na localidade de PC este aumento é significativo (2021 = 6,3 µg/g; IQI = 3,4–13,2; 2023 = 10,4 µg/g; IQI = 6,02–16,35; p = 0,015). CF e TJ também apresentaram um aumento da mediana e IQI das concentrações de arsênio, quando comparados os anos 2021 e 2023, porém sem significância estatística (p > 0,10). Em AR, a distribuição das concentrações de arsênio nos três anos analisados não mostrou variação estatística (p = 0,574) (Tabela 5).

**Tabela 5:** Distribuição das concentrações urinárias de metais da população de estudo por localidade e ano. Projeto Bruminha, 2021, 2022 e 2023.

Local	Metal	2021	2022	2023	P valor
		Mediana (IQI)	Mediana (IQI)	Mediana (IQI)	
Total	As (µg/g)	9,35 (5,45–13,9)	8,6 (5,1 –13,3)	10,8 (7,0–15,3)	**0,064**
	Cd (µg/g)	<LOD	<LOD	<LOD (<LOD–0,1)	0,001
	Hg (µg/g)	0,30 (0,18–0,83)	<LOD (<LOD–0,7)	0,20 (0,1–0,3)	0,008
	Mn (µg/L)	0,35 (0,2–0,68)	0,4 (<LOD–0,8)	0,3 (0,3–0,9)	0,497
	Pb (µg/L)	0,8 (0,4–1,3)	<LOD (<LOD–0,5)	0,56 (0,3–0,9)	0,000
	**Metal**	**Mediana (IQI)**	**Mediana (IQI)**	**Mediana (IQI)**	
Aranha	As (µg/g)	10,40 (6,38–15,4)	9,8 (5,7–14,2)	10,70 (6.65–14.95)	0,574
	Cd (µg/g)	<LOD	<LOD	0,10 (<LOD–0.10)	0,003
	Hg (µg/g)	0,30 (0,20– 0,55)	0,1 (<LOD–0,53)	0,20 (0,1–0,3)	0,413
	Mn (µg/L)	0,40 (0,2– 0,7)	0,5 (<LOD–0,78)	<LOD	0,009
	Pb (µg/L)	0,70 (0,40–1,20)	0, 5 (0,13–0,8)	0,45 (0,28–0,80)	**0,000**
	**Metal**	**Mediana (IQI)**	**Mediana (IQI)**	**Mediana (IQI)**	
Parque da Cachoeira	As (µg/g)	6,3 (3,4–13,2)	8,65 (5,4–16,2)	10,40 (6,02–16,35)	**0,015**
	Cd (µg/g)	<LOD	< LOD (<LOD–0,2)	<LOD (<LOD–0,10)	0,05
	Hg (µg/g)	1,30 (0,15–1,3)	<LOD (<LOD–0,25)	0,20 (0,10–0,30)	0,164
	Mn (µg/L)	0,20 (0,1–0,3)	0,45 (0,13–1,02)	0,55 (0,3–1,18)	**0,000**
	Pb (µg/L)	0,60 (0,33–0,98)	< LOD (<LOD–0,1)	0,85 (0,39–1,34)	0,000
	**Metal**	**Mediana (IQI)**	**Mediana (IQI)**	**Mediana (IQI)**	
Córrego do Feijão	As (µg/g)	8,60 (3,5–8,6)	5,4 (2,95– 9,8)	11,30 (7,1–22,15)	0,112
	Cd (µg/g)	<LOD	<LOD	-	0,528
	Hg (µg/g)	0,2 (<LOD–0,83)	0,1 (<LOD–0,1)	0,18 (0,1– 0,25)	0,274
	Mn (µg/L)	0,5 (0,23–0,7)	<LOD	-	0,000
	Pb (µg/L)	0,75 (0,43–1,18)	<LOD	0,32 (0,145–0,67)	0,000
	**Metal**	**Mediana (IQI)**	**Mediana (IQI)**	**Mediana (IQI)**	
Tejuco	As (µg/g)	9,20 (5,48–12,7)	7,5 (4,1–11,1)	12,00 (6,9–13,0)	0,318
	Cd (µg/g)	<LOD	<LOD (<LOD–0,2)	0,10 (<LOD–0,1)	0,098
	Hg (µg/g)	0,2 (0,2–0,9)	<LOD (<LOD–0,2)	0,20 (0,1–0,3)	0,077
	Mn (µg/L)	0,4 (0,2–0,9)	0,7 (0,3–1,0)	0,60 (0,5–0,9)	0,435
	Pb (µg/L)	1,15 (0,1–1,6)	<LOD (<LOD–0,2)	0,61 (0,4–0,87)	0,000

As: arsênio; Cd: cádmio; Hg: mercúrio; Mn: manganês; Pb: chumbo. LOD: limite de detecção; IQI: intervalo interquartil.

P-valor: em negrito os valores com significância estatística.

Apesar do aumento da taxa de detecção do chumbo urinário no ano de 2023 (100%), não há uma variação significativa do intervalo de distribuição e da mediana das concentrações, no total e em todas as localidades, exceto AR, onde observa-se uma diminuição significativa (p = 0,000) na mediana das concentrações ao longo dos três anos (2021 = 0,70 µg/L; 2022 = 0,50 µg/L; 2023 = 0,45 µg/L). Em relação ao mercúrio e cádmio, o aumento da taxa de detecção observado no ano de 2023 não alterou as medianas desses metais na urina. Observou-se um aumento significativo (p = 0,000) na mediana e IQI para o manganês apenas na localidade de PC ao longo dos três anos, porém mantendo-se abaixo do VR.

## DISCUSSÃO

Os resultados deste estudo mostraram um aumento da exposição infantil aos resíduos dos metais avaliados, com maiores taxas de detecção encontradas no ano de 2023, em relação ao ano de 2021. Arsênio foi detectado em 100% das urinas analisadas nos três anos do Projeto, enquanto as taxas de detecção de chumbo, mercúrio e cádmio subiram de 88,9%, 63,9% e 23,2% em 2021, para 100%, 100% e 48,8% em 2023, respectivamente.

No entanto, ainda não é possível estabelecer uma tendência de exposição a maior concentração de resíduos de chumbo, mercúrio e cádmio, devido à variação observada na análise dos três anos. No ano de 2022, 25% das amostras analisadas, no total e por localidade, estavam abaixo do limite de detecção do método, tornando precoce estabelecer essa tendência. Nos três anos, ocorreu uma diminuição significativa na mediana das concentrações de chumbo na localidade de AR, e os metais restantes permaneceram abaixo dos VR utilizados.

No entanto, em relação as concentrações de arsênio, observa-se um aumento do percentual total de crianças com concentrações urinárias acima do VR ao longo dos três anos (2021 = 42%; 2022 = 44%; 2023 = 57%), e também nas localidades próximas a área do desastre (PC e CF) e de mineração ativa (TJ), em 2023, em comparação com 2021. Nos três anos, as medianas e intervalos de distribuição das concentrações desse metal aumentaram na população total (p = 0,064) e, de forma significativa, nas crianças residentes no PC (p = 0,015), sugerindo uma tendência de maior exposição a este metal.

De forma geral, os resultados sugerem uma disseminação mais ampla desses resíduos no ambiente e, consequentemente, maior número de crianças expostas. Isso pode ser devido ao retorno da atividade minerária na região, principalmente a partir do ano de 2022, que estava interrompida após o desastre e durante a pandemia de covid-19. Essa maior disseminação de resíduos pode estar associada ao aumento dos relatos de alterações respiratórias.

Antes do desastre, as principais fontes de abastecimento de água para consumo humano referidas em todas as localidades eram cisternas coletivas e poços ou nascentes, configurando uma condição de vulnerabilidade socioambiental^13^. Após o desastre, a mineradora responsável começou a fornecer água mineral para a população moradora das localidades impactadas, incluindo TJ, que é uma comunidade situada muito próxima, e em nível inferior, a uma extensa área de mineração que está novamente ativa. PC e CF estão situadas no perímetro das atividades de remediação em andamento. Além da possibilidade de contaminação do lençol freático, é importante assinalar a possibilidade de inalação da poeira decorrente destas atividades.

O termo “níveis basais de exposição” (*background exposure*), indica as concentrações das substâncias químicas às quais a população em geral está exposta, a partir da presença destas substâncias no ambiente (ar, água, solo, alimentos, poeiras etc.)^14^. Em vários países têm sido realizados estudos que indicam os níveis basais de exposição ambiental a metais em suas populações^15^. No período de 2017–2018, o *National Report on Human Exposure to Environmental Chemicals*, parte do *National Health and Nutrition Survey* dos Estados Unidos da America^18^, identificou em crianças entre três e cinco anos uma mediana para arsênio urinário de 8.19 µg/g (7.54–8.95) com um 95 percentil de 42.4 µg/g (27.8–70.6), para chumbo urinário de 0.481 µg/g (0.444 – 0.538) e 95 percentil de 1.65 µg/g (1.24–2.31), e para mercúrio urinário um 95 percentil de 0.390 µg/g (0.210–1.19), com mediana abaixo do limite de detecção. No Projeto Bruminha, em relação às concentrações de arsênio ao longo dos três anos, as medianas estão acima (2021 = 9,35 µg/g; 2022 = 8,6 µg/g; 2023 = 10,85 µg/g), e os percentis 95 estão abaixo (2021 = 27,01 µg/g; 2022 = 24,6 µg/g; 2023 = 25,66 µg/g), dos valores dos Estados Unidos. As medianas e 95 percentis das concentrações de chumbo e de mercúrio urinários estão acima dos valores dos Estados Unidos, exceto no ano de 2022.

No entanto, comparações entre diferentes populações podem ter erros, devido à variabilidade das características e condições econômicas, sociais, culturais, padrões de alimentação, de contato e interação com o ambiente, processos de produção existentes (indústrias etc.), características do ambiente (geológicas, climáticas, hidrológicas, urbanização etc.) no local/área/região que podem levar a diferentes níveis de exposição da população que ali vive. Outro ponto que torna as comparações entre as populações difícil é o período (tempo) no qual o estudo foi realizado. A exposição a químicos ambientais sofre alterações ao longo do tempo, podendo serem observadas variações nos padrões de exposição em uma mesma população, em diferentes anos.

Observa-se uma redução significativa do risco de atraso no neurodesenvolvimento na população estudada ao longo dos três anos. O melhor desempenho das crianças observado na realização das tarefas associadas com o desenvolvimento das habilidades de linguagem, cognitivas e motoras, pode ser justificado pelo retorno das atividades escolares e da vida social das crianças, após o isolamento provocado pela pandemia de covid-19^19^. No entanto, é importante observar que essa melhora foi significativamente maior nas crianças vivendo nas localidades impactadas pelo desastre. Antes do isolamento decorrente da pandemia de covid-19, as crianças dessas localidades já vivenciavam uma situação de anormalidade social em seu ambiente familiar, escolar e comunitário. Alterações das trajetórias de vida repentinas, sofrimento emocional, percepção de desarranjo e insegurança pessoal e comunitária já constituíam o pano de fundo social destas famílias durante o período pré-pandemia. É possível supor que o progressivo retorno à normalidade social após 2022 tenha favorecido uma recuperação mais expressiva dessas crianças, embora o aumento da escolaridade materna e renda familiar, ao longo dos anos, também possam estar relacionadas.

O término das medidas restritivas de socialização também pode estar associado com a melhoria do padrão de avaliação antropométrica observado na população estudada, com a possibilidade de brincadeiras ao ar livre e prática de esportes.

Este estudo apresenta como limitação a aferição das informações de alterações respiratórias a partir de relatos dos pais ou responsáveis, sem validação nos registros de prontuários médicos. Da mesma forma, o teste de Denver se constitui em um teste de triagem, cujo resultado indica uma suspeita de alteração do neurodesenvolvimento, a qual deve ser confirmada por meio de uma avaliação clínica acurada.

É importante enfatizar que ao longo dos três anos não se observam associações entre a exposição aos resíduos de minério e a ocorrência de quaisquer dos desfechos estudados^20^. O Projeto Bruminha não busca o estabelecimento de nexo de causalidade. Nos casos de exposição de longo prazo e baixas doses, como neste estudo, o efeito tóxico das substâncias encontradas pode se manifestar de forma ampla e tardia, desencadeando ou agravando processos mórbidos que apresentam etiologia multicausal.

É por essa razão que é fundamental que o SUS também organize suas diretrizes e ações pelas especificidades dos processos produtivos existentes nos territórios. Em regiões como o quadrilátero ferrífero de Minas Gerais, em que o processo produtivo determinante é a atividade de mineração, a qual, *per si,* é geradora de resíduos para o ambiente e, consequentemente, exposição da população que vive nesse ambiente, é fundamental uma vigilância em saúde ambiental que articule ações entre as secretarias (municipais e estaduais) de saúde e ambiente, com o objetivo precípuo de avaliar a exposição humana aos resíduos. Da mesma forma, as equipes de saúde das unidades de atenção básica, coordenadoras do cuidado à saúde da população adscrita, devem ser capacitadas sobre os potenciais efeitos tóxicos desses resíduos sobre a saúde humana, grupos populacionais vulneráveis e aplicação de protocolos de monitoramento, investigação e assistência, incluindo a possibilidade de requisição de exames toxicológicos específicos, bem como o estabelecimento da rede de atenção à saúde por meio de equipes multiprofissionais no nível primário (antigos –Núcleos Ampliados de Saúde da Família), nível secundário e nível terciário de complexidade, com participação de especialistas e instituições com *expertise* na área.

Os resultados descritos anteriormente mostram um cenário de exposição permanente da população de estudo do Projeto Bruminha a resíduos de metais potencialmente tóxicos à saúde infantil, sem indicação de alterações de saúde associadas aos mesmos, no momento de realização do estudo. Todo e qualquer processo de adoecimento é consequente não apenas da exposição a agentes químicos, físicos ou biológicos, unicausais, mas, também, do arcabouço de condições econômicas, sociais, culturais, genéticas e ambientais que estruturam a percepção e a condição de saúde dos indivíduos. Adicionalmente, em se tratando da população infantil, o estado nutricional, de desenvolvimento, a capacidade de metabolização e defesa, e o grau de maturidade do organismo, são fatores determinantes da relação entre saúde e doença.

Esse cenário indica que é primordial a articulação das equipes e gestores da atenção básica em saúde com os setores de vigilância ambiental e sanitária do Município, Estado e governo federal, para a construção de um SUS que atenda as especificidades dos perfis produtivos dos territórios de abrangência.

## Data Availability

Como a coorte está em desenvolvimento, o banco de dados ainda não está completo e portanto, não disponível no momento.
